# Examination of Amorphous Calcium Carbonate on the Inflammatory and Muscle Damage Response in Experienced Resistance Trained Individuals

**DOI:** 10.3390/nu14091894

**Published:** 2022-04-30

**Authors:** Jay R. Hoffman, Tavor Ben-Zeev, Amit Zamir, Chagai Levi, Ishay Ostfeld

**Affiliations:** Department of Physical Therapy, School of Health Sciences, Ariel University, Ariel 40700, Israel; tavorbenzeev@gmail.com (T.B.-Z.); amitza@ariel.ac.il (A.Z.); c.ashraf5@gmail.com (C.L.); iostfeld@hotmail.com (I.O.)

**Keywords:** ergogenic effects, dietary supplementation, performance, recovery, exercise

## Abstract

The effect of 3 weeks of amorphous calcium carbonate (ACC) supplementation (2000 mg per day) was examined on the recovery response to resistance exercise. Thirty men were randomized into a supplement (ACC) or placebo (PL) group. Following supplementation, participants performed six sets of 10 repetitions in the bench press (BP) and incline BP exercises, using 80% of maximal strength. Participants returned 24 (T4) and 48 h (T5) later and performed six sets of the BP exercise. Significant decreases in the number of repetitions (*p* < 0.001), peak power (*p* < 0.001), and mean power (*p* = 0.009) were noted over time, but no significant interactions were observed (*p* > 0.05). Magnitude-based inference analysis (MBI) indicated that the change in repetitions was possibly beneficial for ACC at T4 and likely beneficial at T5. No significant interaction was noted for general soreness (*p* = 0.452), but a trend toward an interaction was observed in upper body soreness (*p* = 0.089). Confidence intervals for mean percent change scores indicated significant differences between the groups at T4 and T5, and MBI analysis indicated that ACC was very likely or likely to be beneficial for reducing soreness at those time points. In conclusion, ACC supplementation may have a potential beneficial effect in attenuating the decline in performance, which is possibly due to the carbonate component.

## 1. Introduction

Calcium is a micronutrient that has an important role in both bone health and skeletal muscle function [1]. Calcium is an integral part of bone composition and is involved in muscle regulation via its critical role in skeletal muscle contraction and relaxation [2]. Calcium supplementation is often used to achieve adequate calcium intake. Several meta-analyses have concluded that calcium supplementation is effective at enhancing bone strength, but there is some thought that it may be more efficacious when it is combined with vitamin D [3,4]. When provided by itself, its efficacy for enhancing bone strength has been inconclusive [5,6,7]. Research examining the efficacy of calcium supplementation and muscle performance in young, athletic populations is limited. Most studies have generally focused on an older population and often in conjunction with vitamin D ingestion. 

Calcium supplements can be divided into either an organic or inorganic group, which differ on their chelating capability [8]. Organic calcium supplements include negatively charged organic molecules, such as malate, citrate, fumarate, and gluconate, while inorganic calcium supplements include carbonates, phosphates, and chlorides [8]. The calcium found in most dietary supplements is from either carbonate and citrate salts derived from various sources including oyster shells, coral calcium, dolomite minerals, and other synthetic material [9]. The limited absorbability of calcium from these sources is thought to contribute to the lack of consistency reported regarding the efficacy of calcium supplementation [10]. Calcium carbonate is one of the most abundant minerals in nature, having six known polymorphs. The most stable form of calcium carbonate is calcite, while the least stable polymorph is the amorphous form [11]. However, amorphous calcium carbonate is reported to have a significantly greater absorption capability than the calcite form [12]. 

The shell of freshwater crayfish is constructed with a specialized transient mineral storage site (i.e., gastroliths), which is composed of stabilized amorphous calcium carbonate embedded into an organic matrix comprising dense chitin fibers and proteins [13]. The instability of the amorphous calcium carbonate provides a highly bioavailable calcium source that enables a fast and effective transport of the mineral across the intestinal epithelium and into the hardening exoskeleton of the crayfish. This biological finding resulted in the development of a novel method for the synthetic production of stabilized amorphous calcium carbonate using phospho-amino acids [8]. The superior bioavailability of the amorphous calcium carbonate over crystalline calcium carbonate has been demonstrated in both animal [14] and human studies [15]. 

It has been well-established that resistance exercise can result in varying degrees of muscle damage, resulting in an inflammatory response and performance impairments [16]. There have been a number of dietary supplements that have been shown to accelerate recovery from exercise [16]. These supplements may allow an accumulation of specific nutrients within the tissues of the body or in the circulation that will enhance the athlete’s ability to recover. The greater absorption capability of the amorphous calcium carbonate (ACC) would potentially provide for a quicker and more efficient buffering capability of hydrogen ions (H^+^) during high-intensity exercise. In consideration of the limited research on young, active participants, the purpose of this study was to examine the effect of supplementing with ACC on the recovery response to intense resistance exercise in resistance trained men.

## 2. Materials and Methods

### 2.1. Participants 

Thirty experienced, resistance-trained men volunteered to participate in this study. Participants were randomly divided into a supplement (ACC; *n* = 15, age = 25.5 ± 4.4 years old, height = 175.9 ± 6.8 cm, body mass = 84.2 ± 17.3 kg) or a placebo (PL; *n* = 15, age = 26.6 ± 3.1 years old, height = 179.6 ± 8.4 cm, body mass = 89.1 ± 16.3 kg) group. A simple 1:1 randomization procedure was employed. All participants had at least 1-year of resistance training experience (mean 5.8 ± 3.3 years old), including specific experience in performing the bench press exercise. Following an explanation of all procedures, including the risks and benefits associated with volunteering for the study, each participant provided his informed consent. The Institutional Review Board of Ariel University (AU-HEA-JH-20210603) approved the research protocol. Participants were not permitted to use any additional nutritional supplements for at least six weeks prior to the study and did not consume anabolic steroids or any other anabolic agents known to enhance performance for the previous year. Screening for supplement and steroid use was accomplished via a health history questionnaire completed during the recruitment phase.

### 2.2. Study Protocol

The investigation was performed as a double-blind, randomized design. Participants reported to the Human Performance Laboratory (HPL) on five separate occasions. On the first visit (T1), participants received an explanation of the study, provided their informed consent, and were randomized into either the ACC or PL groups. Participants commenced supplementation at T1. Two weeks later, the participants reported to the HPL again (T2) for maximal strength (one repetition-maximum (1-RM)) assessments on the bench press and incline bench press exercises. On their third visit (T3), 7 days following T2, participants performed an upper body resistance exercise session which consisted of six sets of the bench press and incline bench press exercises. The rest interval between each set was 60 s. Each set was performed with 80% of the participant’s previously measured 1-RM. This protocol was based on simulating a typical training program during the hypertrophy phase of training [17], and it has been previously used in other studies examining the effect of nutrient intervention on recovery during high-intensity exercise [18,19]. Participants were required to perform no more than 10 repetitions for each set. No forced repetitions were performed, and resistance was not lowered for subsequent sets if the participant was unable to perform the required number of repetitions in the previous set. Participants reported back to the HPL 24 (T4) and 48 h (T5) post-exercise. During T4 and T5, participants performed six sets of the bench press exercise only using the same loading pattern and rest interval length as T3. The bench press and incline bench press exercises were selected due to the participants’ familiarity with this exercise and years of experience performing it. Any changes noted would be considered the result of the study protocol and not related to any physiological adaptation stimulated by the study itself.

### 2.3. Supplement Protocol

Participants consumed either 2000 mg of ACC or placebo per day. The supplement or placebo was consumed four times per day (500 mg per serving) for 21 days. Calcium comprised 32% of the supplement [15], and the total calcium intake was similar to that used in most calcium supplement studies [3]. During each serving, the participant consumed two packets of the powder. The powder was placed under the participants’ tongues and dissolved sublingually. There was no difference in the appearance or taste between the active ingredient and placebo. Each participant consumed one serving of the supplement 30 min prior to each resistance training session.

### 2.4. Maximal Strength Testing

The 1-RM tests were performed using methods previously described [20]. Each participant performed two warm-up sets using a resistance that was approximately 40–60% and 60–80% of their estimated 1-RM, respectively. The third set was the first attempt at the participant’s 1-RM. If the set was successfully completed, then weight was added, and another set was attempted. If the set was not successfully completed, then the weight was reduced, and another set was attempted. A 3–5-min rest period was provided between each set. The process of adding and removing weight was continued until a 1-RM was reached. Attempts that did not meet the range of motion criterion for each exercise, as determined by the researcher, were discarded. The participants were required to lower the bar to their chest before initiating the concentric movement. Grip widths were measured and recorded for later use.

### 2.5. Performance Measures

Upper body power during the bench press exercise protocol was measured for each repetition with a Tendo™ Power Output Unit (Tendo Sports Machines, Trencin, Slovakia). The Tendo™ unit consists of a transducer that is attached to the end of the barbell, which measures linear displacement and time. Subsequently, bar velocity was calculated, and power was determined. Both peak and mean power output were recorded for each repetition and used for subsequent analysis. Test–retest reliability for the Tendo unit has been shown to be R > 0.90 [18,21].

### 2.6. Soreness Questionnaire

To provide a subjective measure of the participants’ perceptions of muscle and whole-body soreness, participants were asked to rate their degree of whole-body muscle soreness and upper-body muscle soreness at T3, T4, and T5 using a 15 cm visual analog scale (VAS). Participants were asked to rate their feelings of soreness by marking on a line with words anchored at each end of the VAS. Questions were structured as “My level of muscle soreness is:” with the words “low” and “high” serving as the verbal anchors representing the extreme ratings. Therefore, the greater the measured value, the greater the feeling. The VAS was conducted following each blood draw prior to performing the workouts on each assessment day. The validity and reliability of VAS in assessing fatigue and energy has been previously established [22].

### 2.7. Blood Measurements

During the T3 experimental session baseline (PRE), blood samples were obtained prior to exercise. An additional blood sample was drawn 60 min post-exercise (POST). All blood samples were obtained using a 20-gauge Teflon cannula placed in a superficial forearm vein. The cannula was maintained patent using an isotonic saline solution (with 10% heparin). PRE blood samples were drawn following a 15-min equilibration period prior to exercise. All T3 blood samples were obtained while the participant was in a seated position. During the T4 and T5 sessions, only a baseline blood sample was drawn. These blood samples were obtained from an antecubital arm vein using a 20-gauge disposable needle equipped with a Vacutainer^®^ tube holder with the participant in a seated position. Each participant’s blood samples were obtained at the same time of day during each session. Blood samples were collected into a single Vacutainer^®^ tube, containing SST^®^ Gel and Clot Activator. The blood was allowed to clot at room temperature and subsequently centrifuged at 1500× *g* for 15 min. The resulting serum was placed into separate 1.8 mL microcentrifuge tubes and frozen at −80 °C for later analysis. All blood draws were conducted by individuals trained in phlebotomy.

### 2.8. Biochemical Analyses

Serum concentrations of creatine kinase muscle (CK-M) were analyzed with enzyme-linked immunosorbent assay (ELISA) kits per manufacturer’s instructions. CK-M was analyzed using kits from Abcom (ab264617, San Diego, CA, USA). Serum concentrations of pro- and anti-inflammatory cytokines including tumor necrosis factor-alpha (TNF-α), interleukin (IL)-6, and IL-10 were analyzed via multiplex assay using Human Cytokine/Chemokine Panel I (EMD Millipore, Billerica, MA, USA). All samples were thawed once and analyzed in duplicate by the same technician using an Absorbance 96 spectrophotometer (Byonoy GmbH, Hamburg, Germany) for the CK-M assay and MagPix (EMD Millipore) for cytokine concentrations. The mean intra-assay variability for all assays was <10%.

### 2.9. Statistical Analysis

Prior to analysis, all data were assessed to ensure normal distribution, homogeneity of variance, and sphericity. If sphericity was violated, a Greenhouse–Geisser correction was applied. Statistical evaluation of performance and biochemical changes was accomplished using a two-way (group × time) repeated analysis of variance (ANOVA). If there was a significant main effect for time, then the post hoc analysis for each group was assessed using 95% confidence intervals (CI) as previously described [23,24]. Percent change scores were calculated for each participant from T3 to T4 and from T3 to T5 for performance, soreness, and CK-M measures, while percent change scores were calculated for the cytokine measures from PRE to POST, PRE to T4, and PRE to T5. These percent change scores were averaged separately for the ACC and PL groups, and 95% CI were constructed around the mean percent change scores. When the 95% CI included 0, the mean percent-change was considered no different from 0 and was interpreted as no statistical change [25,26]. However, if the 95% CI did not include 0, the mean percent change for that variable was considered statistically significant at *p* ≤ 0.05 [25,26]. In addition, if the CIs between groups did not overlap, it was considered to represent a significant interaction [27]. All statistical analyses were analyzed using SPSS v27 software (SPSS Inc., Chicago, IL), and an alpha level of *p* ≤ 0.05 was used to determine statistical significance. All data are reported as mean ± SD.

To complement our null hypothesis testing and to make inferences about the true effects of the supplement on exercise recovery, data were further analyzed using magnitude-based inferences (MBI) [28]. Several studies have suggested that the MBI analysis is an effective statistical tool for null hypothesis testing to reduce interpretation errors [29,30]. Data were calculated from 90% CI and analyzed as previously described [29]. Differences in the change of the ∆ scores between ACC vs. PL at all time points were analyzed using the p-value from independent t-tests to determine a mechanistic inference utilizing a published spreadsheet [31]. All data are expressed as a mean effect ± SD, with percent chances of a beneficial, trivial, or negative outcome. Qualitative inferences, based on quantitative chances, were assessed as: <1% almost certainly not, 1–5% very unlikely, 5–25% unlikely, 25–75% possibly, 75–95% likely, 95–99% very likely, and >99% almost certainly [30]. If there was a greater than 5% chance that the true value was either greater or lesser, indicating that the CI was overlapping multiple thresholds, the effect was considered to be mechanistically unclear [30]. The smallest non-trivial change, or smallest worthwhile change, was set at 20% of the grand standard deviation for all PRE-values [30].

## 3. Results

Two of the 30 participants (one participant from each group) improved the number of repetitions performed in the bench press exercise from T3 to T4. This was not expected, and it was suggested that maximal effort was not achieved on T3. These participants were removed from the study, and a total of 28 participants were used in the final analysis (14 per group). No differences were seen in 1-RM bench press (*p* = 0.32; 100.0 ± 18.6 kg and 107.9 ± 20.5 kg) or in the number of repetitions performed in the bench press exercise during T3 (*p* = 0.773, 26.7 ± 5.5 and 27.6 ± 8.7) between ACC and PL, respectively. In addition, the total training volume (repetitions x load) for both the bench press and incline bench press exercises performed at T3 were similar (*p* = 0.34) between ACC (3211 ± 648 kg) and PL (3561 ± 1115 kg).

**Figure 1:**
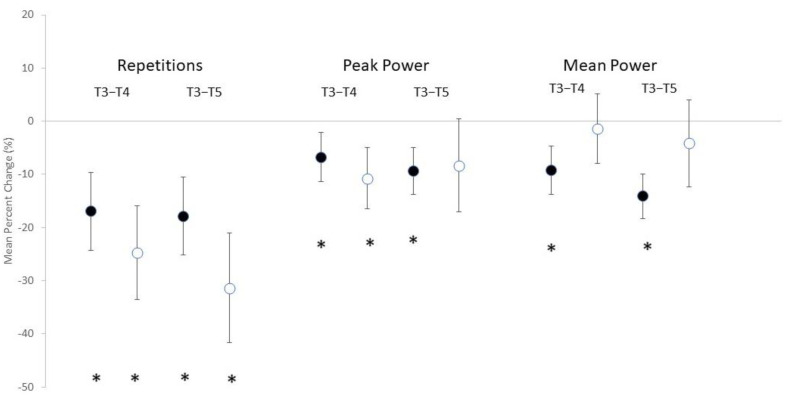
Mean percent change scores ± 95% confidential intervals (CI) for performance valuables. Black circles indicate amorphous calcium carbonate group; white circles represent placebo group. * Indicates a significant difference when 0 is outside of the 95% CI.

The number of repetitions performed per workout in the bench press exercise, the average peak and mean power exhibited for each workout, and subjective feelings of soreness, both general and local, can be seen in Table 1. Significant main effects for time were noted in the number of repetitions performed (F = 27.565, *p* < 0.001), peak power (F = 8.180, *p* < 0.001), and mean power (F = 5.208, *p* = 0.009). However, no significant interactions were noted in the number of repetitions performed (F = 2.077, *p* = 0.136), peak power (F = 0.678, *p* = 0.494), or mean power (F = 2.232, *p* = 0.119). The mean percent change scores (see Figure 1) revealed significant decreases for repetitions performed at T4 and T5 for both ACC and PL. Significant decreases from PRE were noted in peak power for ACC at both T4 and T5, while this was only noted for PL at T4. Similarly, decreases in mean power were noted for ACC at both T4 and T5, but no change was observed for PL. MBI analysis (see Table 2) indicated that the change in repetitions performed was possibly beneficial for ACC compared to PL at T4 and likely beneficial at T5. Similarly, MBI analysis on changes in peak power between ACC and PL indicated that ACC was possibly beneficial for changes in peak power at T4, but it was unclear at T5. This analysis also indicated that ACC supplementation was possibly negative and likely negative for changes in mean power at T4 and T5, respectively.

**Figure 2:**
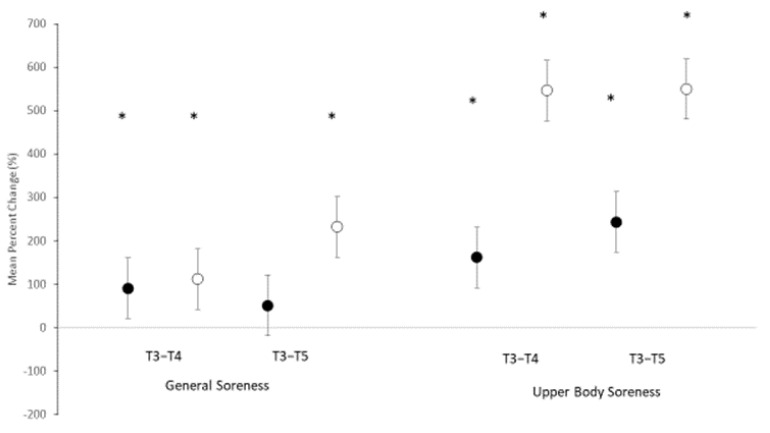
Mean percent change scores ± 95% confidential intervals (CI) for soreness ratings. Black circles indicate amorphous calcium carbonate group; white circles represent placebo group. * Indicates a significant difference when 0 is outside of the 95% CI.

Results for subjective measures of soreness, both general and upper body, are also depicted in Table 1. Significant main effects for time were noted for both general soreness (F = 5.703, *p* = 0.006) and soreness specific for the upper body (F = 26.287, *p* =< 0.001). No significant interaction was noted in general soreness (F = 0.809, *p* = 0.452), but a trend toward an interaction was observed in upper body soreness (F = 2.551, *p* = 0.089). The mean percent change scores (see Figure 2) revealed significant changes from PRE in general soreness for ACC at T4 only, while significant increases in soreness were noted for PL at both T4 and T5. Examination of the CIs for general soreness at T5 and upper body soreness at T4 and T5 clearly show a separation in the change score suggesting a significant difference between the groups. MBI analysis (see Table 2) indicated that ACC was unclear for reducing general body soreness at T4 and at T5. However, when examining the effects of ACC on reducing specifically upper body soreness, results indicated that ACC was very likely (97.4%) or likely beneficial at T4 and T5, respectively.

**Figure 3:**
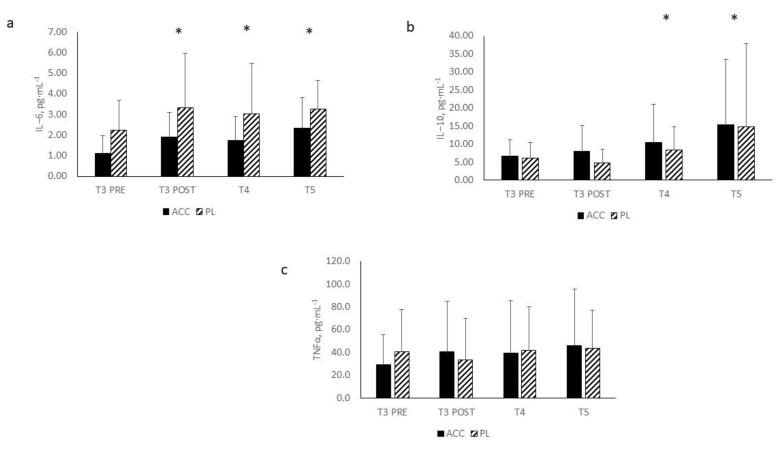
Cytokine response to the exercise protocol: (**a**): IL-6; (**b**): IL-10; (**c**): TNFα. All data are presented as mean ± SD. IL-6 = interleukin-6; IL-10 = interleukin-10; TNFα = Tumor necrosis factor alpha; * = significant main effect for both groups combined versus PRE.

**Figure 4:**
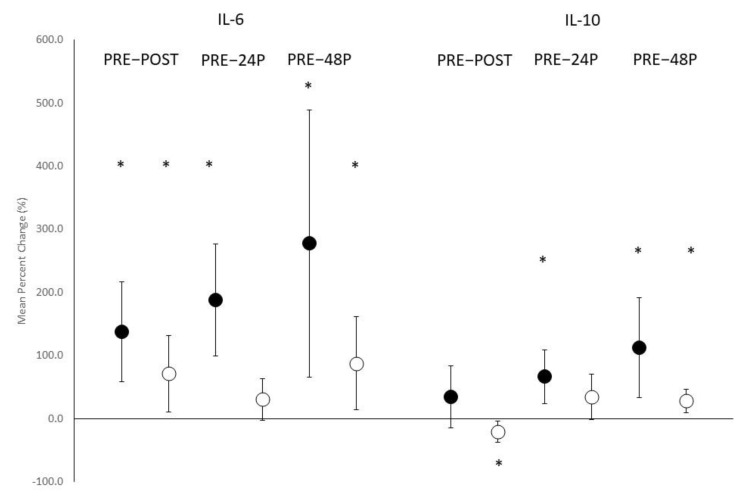
Mean percent change scores ± 95% confidential intervals (CI) for IL-6 and IL-10 responses. Black circles indicate amorphous calcium carbonate group; white circles represent placebo group. * Indicates a significant difference when 0 is outside of the 95% CI.

Changes in cytokine concentrations are depicted in Figure 3a–c. Significant main effects for time were observed in IL-6 (F = 4.058, *p* = 0.017) and IL-10 (F = 4.128, *p* = 0.048) but not TNFα (F =2.025, *p* = 0.138). With both groups combined, significant elevations in IL-6 concentrations were noted from PRE at POST (*p* = 0.005), T4 (*p* = 0.019), and T5 (*p* = 0.004), while significant elevations were noted in IL-10 concentrations from PRE at T4 (*p* = 0.022) and T5 (*p* = 0.046). No significant interactions were noted between ACC and PL in circulating concentrations of IL-6 (F = 0.201, *p* = 0.859), IL-10 (F = 0.103, *p* = 0.786), or TNFα (F =1.705, *p* = 0.190). Mean percent change scores (see Figure 4) indicated significant elevations in percent change for IL-6 concentrations from PRE at POST and T5 for both ACC and PL but only for ACC at T4. Examination of percent changes in IL-10 concentrations resulted in a significant decrease in IL-10 from PRE to POST for PL only, where a significant elevation was noted at T4 for ACC only. Significant elevations in percent changes were noted for both ACC and PL at T5. MBI analyses for the changes in cytokine concentrations from PRE (see Table 3) revealed that differences between ACC and PL in the IL-6 response were unclear for all time comparisons. Changes from PRE in IL-10 concentrations was likely beneficial for ACC compared to PL at POST. The benefits of ACC supplementation on changes in IL-10 concentrations at T4 and T5 were unclear. ACC supplementation appeared to be likely negative, possibly negative, and likely negative for changes in TNFα concentrations at POST, T4, and T5, respectively.

**Figure 5:**
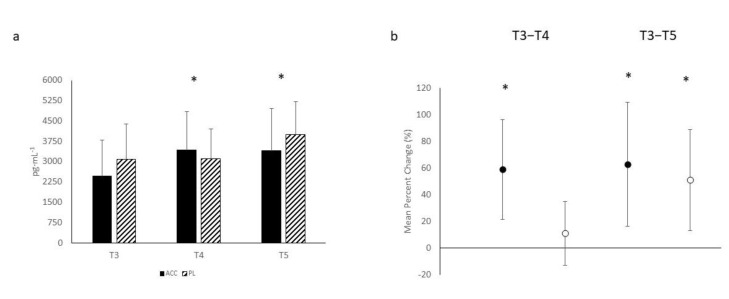
(**a**): Creatine kinase-muscle (CK-M) response to the exercise protocol; (**b**): Mean percent change scores ± 95% confidential intervals (CI) for CK-M response. Black circles indicate amorphous calcium carbonate group; white circles represent placebo group. * Indicates a significant difference when 0 is outside of the 95% CI.

Results for changes in creatine kinase concentrations can be observed in Figure 5a. A significant main effect for time (F = 5.580, *p* = 0.007) was seen, but no significant interaction was noted between the groups (F = 1.914, *p* = 0.160). Mean percent change scores (see Figure 5b) showed significant differences between T3 and T4 for ACC only, but for both ACC and PL between T3 and T5. MBI analysis (see Table 3) indicated that the change from T3 at T4 was very likely negative for ACC and unclear at T5.

## 4. Discussion

The aim of this study was to determine whether 3 weeks of ACC supplementation can enhance recovery from consecutive days of resistance training. The results from the performance measures, subjective feelings of soreness and blood markers of muscle damage and inflammation, appear to be inconclusive. Parametric analysis was unable to provide traditional statistical support regarding ACC supplementation and enhanced recovery. However, complementary analyses using confidential intervals and inferential analysis were suggestive of possible and likely benefits in performance improvements in consecutive days of exercise in participants consuming ACC. Furthermore, confidence interval examination of percent change scores indicated that the differences in soreness between the groups was significant. More specifically, participants in ACC had less soreness in the upper body than PL at both T4 and T5, which was supported by inferential analysis showing that ACC was very likely and likely beneficial at T4 and T5, respectively. Changes in the cytokine and CK-M measures though were not suggestive of any clear benefit for ACC supplementation on reducing inflammation or muscle damage.

Calcium supplementation has generally not been associated with being ergogenic [1]. However, the greater absorbability and stability of amorphous calcium stimulated the curiosity that this specific calcium supplement can increase the likelihood of an ergogenic effect. Considering that most of the body’s calcium is stored in bone [32], its role in muscle performance is not well-understood. During high volume activity in which calcium levels may fall, athletes may be at greater risk for fractures, which has been shown to be reversed with calcium supplementation [33]. Although the inferential analysis performed in this study suggested that ACC supplementation may be possibly or likely beneficial for attenuating performance decrements, the mechanism that underlies this response is not clear. We believe that the difference in the number of repetitions performed at T4 (35%) and T5 (55%) between ACC and PL is likely related to the carbonate component of the supplement. Carbonate has a known ergogenic effect by buffering increases in hydrogen ions produced during high-intensity exercise [34]. Previous research had demonstrated a greater number of repetitions performed in the back squat but not the bench press exercise following an acute ingestion of sodium bicarbonate [35]. Although participants in the present study supplemented for 3 weeks, they were also instructed to consume a dose 30 min prior to each exercise bout. Thus, it is not clear whether the benefits associated from this study were related to the prolonged supplementation schedule or the acute dose provided prior to the exercise protocol.

Supplementation with ACC appeared to result in a negative response for mean power. This was likely related to the greater number of repetitions performed by ACC. Considering that the carbonate component may have increased resiliency to the fatiguing nature of the exercise, the additional repetitions performed resulted in a lower mean power output leading to the appearance of negative effect. This is supported by recent research that indicated a dose–response effect between repetitions performed and power output [36]. When examining the peak power performance, inferential analysis suggested that ACC supplementation provided a possible benefit at T4.

The reduced soreness ratings specific to the upper body suggested that ACC supplementation likely or very likely provided a benefit. This was noted in both the MBI analysis as well as the 95% CI measurements. The mechanism associated with this finding, however, can only be speculative. However, pain perception during or following exercise is thought to be a result in part to increases in proton concentrations [37]. It is possible that the carbonate molecule may have reduced upper body muscle soreness due to its buffering effect. Previous research has reported that an increase in membrane permeability from exercise-induced muscle damage results in a disruption to the transverse tubule (T-tubule) system impacting excitation–contraction coupling [38]. Damage to the T-tubule system directly impacts the sarcoplasmic reticulum, storage site of calcium, resulting in uncontrolled calcium entry into the sarcoplasm and a fall in active tension [39]. Elevations in intracellular calcium have also been demonstrated to activate the calpain proteolytic pathway, resulting in the enzymatic degradation of cytoskeletal proteins [38,40]. This is not a mechanism for reducing muscle soreness, and whether exogenous calcium intake can reverse this process has not been examined. One case study examining the effect of exogenous calcium ingestion in a patient with sporadic idiopathic hypoparathyroidism reported that calcium supplementation was able to reverse impaired nerve conduction velocity [41]. However, these findings have not been confirmed by any other study. Improvements in muscle strength have been reported from a 6-month study examining the effect of combined calcium and vitamin D administration in vitamin D-deficient individuals [42]. However, the investigators were unable to differentiate the individual effects of calcium from vitamin D (i.e., cholecalciferol). Regardless, other studies have failed to provide any evidence that the combination of calcium and cholecalciferol is effective at increasing muscle strength [43,44].

The CK-M and cytokine responses were consistent with other investigations using a similar exercise protocol [18,19,45]. The significant elevations in CK-M at both T4 and T5 in both groups reflect the damage resulting from the exercise protocol. Interestingly, inferential analysis suggested that ACC supplementation was associated with a likely negative effect at T4. This may be related to the greater number of repetitions performed at that time point. Previous research examining the effect of a nutritional intervention on recovery have reported total CK levels being significantly attenuated [18]. This was not seen in the present study, suggesting that ACC supplementation did not reduce the magnitude of muscle damage. This was consistent with the IL-6 and IL-10 responses, in which no between-group differences were noted in any of the cytokine measures. IL-6 has both pro-and anti-inflammatory roles [46]. Elevations in IL-6 are reported to stimulate the anti-inflammatory cytokine IL-10 [47] and inhibit the production of TNFα [48]. Inferential analysis suggested a benefit from ACC supplementation from PRE to POST at T3 for changes in IL-10 concentrations. However, the unclear effects observed at T4 and T5 question the veracity of that response. Furthermore, the negative effects observed in TNFα from ACC supplementation provide further doubt regarding the supplement’s recovery benefits.

The exercise protocol used in this present study did elicit significant declines in performance and significant elevations in muscle soreness, damage, and inflammation. The ACC intervention did appear to provide some benefit toward attenuating the performance and soreness changes. However, there were several limitations associated with this study. The statistical power of this study may not have been adequate. Unfortunately, there were no previous studies available that examined this specific dietary supplement to provide the necessary information to determine appropriate participant number. The research team instead used previous studies that examined a similar research protocol with a nutrient intervention [17,18]. In addition, this study used a single exercise protocol. Previous research examining a calcium supplement reported increases in lower body but not upper body exercise [34]. Differences in performance outcomes between different exercises are likely related to differences in exercise-specific training experience and technique versus a physiological effect. There is no evidence available demonstrating that calcium supplementation can impact lower body differently that upper body musculature. In conclusion, the results of this study suggest that the attenuation in performance decline and soreness was most probably due to the carbonate component of the supplement.

## Figures and Tables

**Table 1:** Performance and Subjective Measures of Soreness at All Testing Sessions.

Variable	Group	T3	T4	T5
Repetitions Performed (#)	ACC	26.7 ± 5.7	22.2 ± 7.3	22.0 ± 7.4
	PL	27.6 ± 9.0	20.7 ± 9.0	18.9 ± 10.1
Peak Power (w)	ACC	323 ± 51	297 ± 48	285 ± 44
	PL	366 ± 86	322 ± 95	333 ± 103
Mean Power (w)	ACC	207 ± 39	187 ± 29	174 ± 24
	PL	230 ± 53	222 ± 59	219 ± 70
VAS—General body soreness (cm)	ACC	3.8 ± 3.8	4.4 ± 3.5	5.1 ± 3.9
	PL	2.0 ± 2.5	3.6 ± 2.8	4.8 ± 3.4
VAS—Upper body soreness (cm)	ACC	2.3 ± 2.5	4.8 ± 3.1	6.1 ± 4.7
	PL	1.3 ± 1.3	7.0 ± 3.9	7.3 ± 3.7

ACC = Amorphous calcium carbonate; PL = Placebo; VAS = Visual analogue scale; # = Number of repetitions performed; w = Watts. Data are reported as mean ± SD.

**Table 2:** Magnitude-Based Inferences on Change Scores in Performance and Subjective Soreness Variables.

Variable	Time	ACC	PL	Mean Difference	% Positive	% Trivial	% Negative	Interpretation
					Percent Chance Greater			
Repetitions Performed (#)	ΔT4	−4.5 ± 4.6	−6.9 ± 5.8	2.4 ± 4.1	67.4	29.4	3.2	Possibly Beneficial
	ΔT5	−4.7 ± 4.7	−8.6 ± 7.3	3.9 ± 4.7	84.8	13.8	1.4	Likely Beneficial
Peak Power (w)	ΔT4	−23.0 ± 34.4	−38.2 ± 47.7	15.0 ± 34.0	51.6	44.4	4.0	Possibly Beneficial
	ΔT5	−32.2 ± 33.9	−28.3 ± 65.1	−3.9 ± 42.0	18.5	51.3	30.2	Unclear
Mean Power (w)	ΔT4	−21.2 ± 24.2	−4.6 ± 34.8	−17.0 ± 24.0	1.8	25.5	72.6	Possibly Negative
	ΔT5	−31.6 ± 24.1	−6.5 ± 39.4	−25.0 ± 26.0	0.6	11.0	88.3	Likely Negative
VAS—General Soreness (cm)	ΔT4	0.6 ± 3.7	1.6 ± 2.2	−1.0 ± 2.6	61.2	29.0	9.8	Unclear
	ΔT5	1.3 ± 3.4	2.8 ± 2.9	−1.5 ± 2.6	74.9	20.1	5.0	Unclear
VAS—Upper Body Soreness (cm)	ΔT4	2.7 ± 3.5	6.3 ± 3.9	−3.6 ± 3.2	97.4	1.7	0.8	Very Likely Beneficial
	ΔT5	4.0 ± 4.3	6.6 ± 3.2	−2.6 ± 3.3	90.9	5.6	3.5	Likely Beneficial

ACC = Amorphous calcium carbonate; PL = Placebo; VAS = Visual analogue scale; # = Number of repetitions performed; w = Watts. Data are reported as mean ± SD.

**Table 3:** Magnitude-Based Inferences on Change Scores in Cytokine Concentrations.

Variable	Time	ACC	PL	Mean Difference	% Positive	% Trivial	% Negative	Interpretation
					Percent Chance Greater			
IL-6 (pg·mL^−1^)	Δ POST − PRE	0.77 ± 0.78	1.09 ± 1.95	−0.32 ± 1.4	54.2	25.6	20.2	Unclear
	Δ T4 − PRE	0.62 ± 1.34	0.8 ± 1.31	−0.18 ± 1.2	45.1	32.3	22.6	Unclear
	Δ T5 − PRE	1.02 ± 1.72	1.02 ± 1.56	0.18 ± 1.4	27.1	26.8	46.0	Unclear
IL-10 (pg·mL^−1^)	Δ POST − PRE	1.36 ± 4.42	−1.41 ± 2.7	2.80 ± 3.1	89.0	9.7	1.3	Likely Beneficial
	Δ T4 − PRE	3.76 ± 7.59	2.12 ± 3.15	1.6 ± 5.0	62.7	22.0	15.3	Unclear
	Δ T5 − PRE	8.81 ± 14.68	8.58 ± 23.32	0.23 ± 17.0	46.9	8.4	44.7	Unclear
TNFα (pg·mL^−1^)	Δ POST − PRE	11.0 ± 24.4	−7.4 ± 18.9	18 ± 20	1.0	10.0	89.0	Likely Negative
	Δ T4 − PRE	9.8 ± 26.1	1.0 ± 14.9	8.8 ± 20	6.9	32.5	60.6	Possibly Negative
	Δ T5 − PRE	16.4 ± 27.9	3.0 ± 14.9	13 ± 21	3.5	20.7	75.9	Likely Negative
CK-M (pg·mL^−1^)	Δ T4 − T3	968 ± 1169	12 ± 1258	960 ± 1100	1.2	8.5	90.2	Very Likely Negative
	Δ T5 − T3	942 ± 1702	908 ± 1659	33 ± 1500	33.3	29.9	36.8	Unclear

ACC = Amorphous calcium carbonate; PL = Placebo; IL-6 = Interluekin-6; IL-10 = Interleukin-10; TNFα = Tumor necrosis factor α; CK-M = Creatine kinase muscle. Data are reported as mean ± SD.

## Data Availability

The data presented in this study are available on request from the corresponding author.
